# Clinical Manifestations Associated with Pain in Nursing Students in Emerging Adulthood with Long COVID

**DOI:** 10.3390/nursrep16090302

**Published:** 2026-08-26

**Authors:** Marisol Ignacio Albino, Yolanda Hernández Ortega, Lorena Chaparro Díaz, María de Lourdes Rico González, Patricia Cruz Bello, Zuleyma González Miguel, Nicolas Santiago González, María Gicela Pérez Hernandez, Ángel Eduardo Bárcenas García, María de Lourdes García Hernández

**Affiliations:** 1 Faculty of Dentistry, Autonomous University of the State of Mexico, Toluca 50180, Mexico; mignacioa@uaemex.mx; 2 Faculty of Nursing and Obstetrics, Autonomous University of the State of Mexico, Toluca 50180, Mexico; yhernandezo@uaemex.mx (Y.H.O.); mlricog@uaemex.mx (M.d.L.R.G.); pcruzb@uaemex.mx (P.C.B.); zgonzalezm@uaemex.mx (Z.G.M.); 3 Departamento de Enfermería, Facultad de Enfermería, Universidad Nacional de Colombia, Sede Bogotá, Bogotá 111321, Colombia; olchaparrod@unal.edu.co; 4 Ixtapaluca Regional High-Speciality Hospital, Mexican Social Security Institute for Wellbeing (IMSS-BIENESTAR), Mexico-Puebla Federal Highway, Km 34.5, Ixtapaluca 56530, Mexico; nicosantiago21@hotmail.com; 5 Faculty of Nursing, University of Colima, Colima 28040, Mexico; ggise_ph@ucol.mx; 6 Faculty of Medicine, Autonomous University of the State of Mexico, Toluca 50180, Mexico; aebarcenasg@uaemex.mx

**Keywords:** Long-COVID, pain, students, nursing

## Abstract

**Objective**: To identify the clinical manifestations associated with pain in nursing students in emerging adulthood with Long COVID (LC). **Methods**: A cross-sectional, descriptive, and analytical study was conducted. The universe comprised 1508 students from a Faculty of Nursing in the State of Mexico. The sample size of 307 participants was calculated using Cochran’s formula (1977) with 95% confidence and a 5% margin of error, with correction for finite populations; participants were invited through an open call and selected non-probabilistically according to inclusion criteria and willingness to participate. Instrument: A questionnaire on post-SARS-CoV-2 health conditions was used, with reliability assessed in a pilot test through internal consistency measures, obtaining a Cronbach’s Alpha = 0.86 and KR-20 = 0.87. It was administered between the third and fourth week following the three-month mark after COVID-19 onset. Descriptive analysis, Fisher’s exact test, and a binary logistic regression model were used, with pain as the dependent variable (1 = yes, 0 = no); statistical significance was set at *p* < 0.05, with a 95% CI. **Results**: 40.4% of students reported pain—(46.6%) males and (39.0%) females—with a significant association between age group and pain (*p* < 0.001). A total of 198 painful clinical manifestations were reported: headache (48.0%), chest oppression (26.8%), and myalgias (23.2%). The model showed that taste alteration (*p* < 0.001) and decreased vision (*p* < 0.001) were associated with a lower likelihood of pain, while gastrointestinal alterations (*p* = 0.025) and fatigue (*p* = 0.048) were associated with a higher likelihood of pain. **Conclusions**: Pain in students with LC is associated with clinical manifestations, mainly gastrointestinal alterations, and fatigue.

## 1. Introduction

In late 2019, coronavirus disease 2019 (COVID-19), caused by SARS-CoV-2, generated an epidemiological impact worldwide. By 23 June 2024, more than 775 million confirmed cases and more than seven million deaths were reported globally [1]. However, these figures likely underestimate the true magnitude of the pandemic due to differences in diagnostic capacity, surveillance systems, and death registration procedures. A systematic analysis estimated 1.6 million deaths during 2020–2023, concentrated within the three years of the pandemic. Incidence and case fatality rates showed geographic and socioeconomic variations. Infection rates were higher in Europe and high-income countries, whereas case fatality was proportionally higher in regions and countries with fewer resources [2].

In Mexico, the COVID-19 pandemic resulted in a high burden of morbidity and mortality. In 2026, 986,284 confirmed cases and 241,080 deaths were reported. Although incidence declined, SARS-CoV-2 continued to circulate in the country, and during this year, the Secretary of Health reported 709 positive cases and 21 deaths from COVID-19 [3]. These figures must be interpreted in the current context as detected and reported cases, not the total infections occurring in the population. Individuals who survived infection continue to experience persistent manifestations, collectively referred to as Long COVID (LC).

COVID-19 presents three phases of disease progression: 1. early infection; 2. pulmonary; and 3. hyperinflammation, with clinical manifestations ranging from mild symptoms and asymptomatic presentation to Acute Respiratory Distress Syndrome and multiorgan failure [4], leaving Long COVID as a sequela [5], defined by the National Academies of Sciences, Engineering, and Medicine in 2025 as a chronic disease associated with SARS-CoV-2 infection, characterized by symptoms or clinical manifestations present at least three months after COVID-19, which may be continuous, recurrent, remitting, or progressive, and may affect one or more organ systems. Clinical manifestations include fatigue, dyspnea, palpitations, myalgias, and chest pain [6], reflecting the persistence of multisystemic sequelae over an extended period. Headache and cognitive impairment have been associated with possible residual organic damage, post-viral syndromes, or autonomic nervous system alterations [7]. Recent studies describe the prevalence of headache and joint, chest, or generalized pain, as well as manifestations similar to fibromyalgia [8], affecting children and adults, regardless of sex, gender, sexual orientation, race, ethnicity, or socioeconomic status. Health status may be exacerbated by pre-existing conditions or generate new alterations. The severity of LC symptoms varies from mild to severe and disabling. Diagnosis is established clinically, and the repercussions may limit the ability to work, study, provide family care, or maintain self-care, with physical and emotional impacts on patients, families, and caregivers [9].

Pain, as a frequent clinical manifestation of LC, is an unpleasant sensory and emotional experience associated with actual or potential tissue damage [10]. Pain is classified into three types: 1. nociceptive, originating from tissue damage; 2. neuropathic, resulting from nervous system injury or disease; and 3. nocioplastic, associated with altered nervous system processing. Some patients may present a combination of these pain types, making diagnosis and treatment more complex [8].

In Spain, in 2020, pain was identified as a frequent and persistent symptom in patients with COVID-19. Membrilla, Corona, and Trigo (2021) reported that headache may be an initial sign that persists throughout the disease [11,12,13]. García (2022) detailed the semiology of pain in patients with coronavirus infection, describing its relevance and need for urgent attention [14]. Sunkersing, through the University College London (2024), reported that pain is present in 26% of symptoms in individuals with Long COVID, including headache, chest pain, and limb discomfort [15]. Diverse clinical presentations of pain have been identified in younger populations, including cutaneous pain [16], as demonstrated in the study by Torres (2021), which identified pleuritic pain due to complications such as spontaneous pneumothorax and persistent musculoskeletal symptoms similar to fibromyalgia [16]. On the other hand, Becerra (2022) described that chronic pain is associated with symptoms of post-traumatic stress disorder [17], while Faro in 2016 noted that sex influences the development of pain, with notable differences between young men and women [18].

Najeeha Talat Iqbal (2025) associates clinical and epidemiological risk factors for LC with age, female sex, obesity, type 2 diabetes mellitus, severe acute SARS-CoV-2 infection, and nutrient deficiency, identifying the presence of antibodies, reactivation of Epstein–Barr virus, ethnicity, and professional practice in the healthcare field, the latter recognized as an epidemiological risk factor [6]. Bai F. mentions that women, advanced age, and active smoking are considered risk factors for developing Long COVID [19].

To date, studies have focused on pediatric and older adult populations. Although various research efforts have also addressed Long COVID manifestations in adolescents and young adults, evidence in the population of nursing students in emerging adulthood aged 18–29 years remains limited [20]. Nursing students experience psychosocial changes upon beginning university studies, become independent from their families, and undergo lifestyle changes, generating academic stress, physical inactivity, and alterations in diet with increased body weight [21,22]. These conditions increase emerging adults’ vulnerability to respiratory and chronic noncommunicable diseases. Nursing students in Mexico receive academic training with practice in hospital and community settings, increasing the risk of exposure to infectious diseases, including COVID-19, which significantly impacts the biological and psychological state of students, manifesting as symptoms of pain, anxiety, depression, post-traumatic stress, and academic uncertainty [23].

In this context, the objective of this research is to identify the clinical manifestations associated with pain in nursing students in emerging adulthood with Long COVID.

## 2. Materials and Methods

This was a descriptive, cross-sectional, and analytical study conducted with a universe of 1508 nursing students in emerging adulthood from a Faculty of Nursing in the State of Mexico.

The sample size was calculated from the universe of 1508 nursing students using Cochran’s 1977 formula $n_{o=\frac{z^{2}*p*q}{e^{2}}}$, with 95% confidence and a 5% margin of error, with correction for finite populations using the formula for a finite population $n=\frac{n_{0}}{1+\frac{\left(n_{0-}1\right)}{Ν}}$ resulting in a total of 307 participants. Students were invited to participate through an open recruitment call to select those meeting the inclusion criteria. Students were selected as they consented to participate using non-probabilistic sampling until 307 participants were obtained. No questionnaires were eliminated or excluded from analysis.

### Flowchart for Selecting the Study Sample

**Figure 1:**
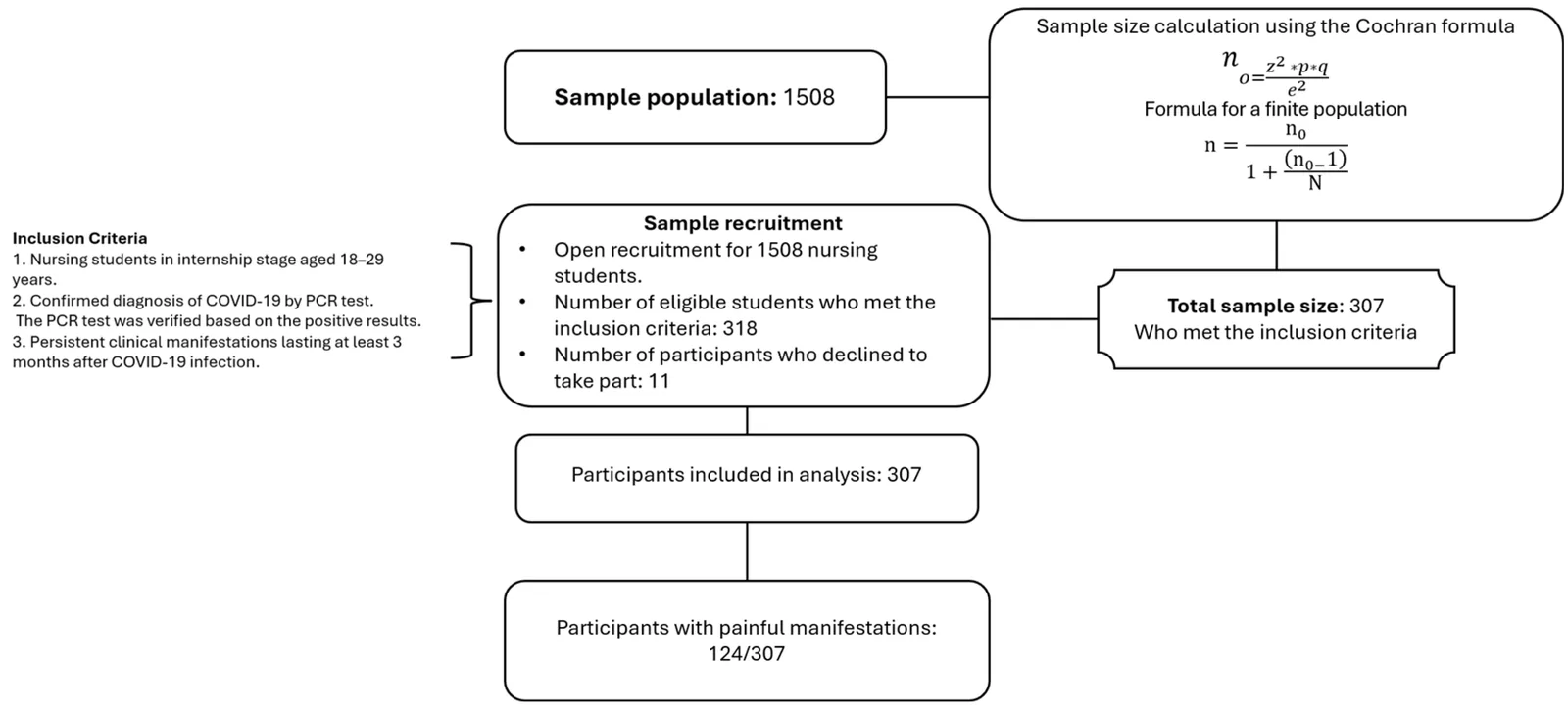
Flowchart for selecting the study sample. Source: Author’s own work, 2026.

Inclusion criteria were: nursing students in emerging adulthood aged 18–29 years with confirmed COVID-19 diagnosis verified by a polymerase chain reaction (PCR) test; PCR test positivity was confirmed by examination of test results; persistent clinical manifestations at least three months after COVID-19 infection [9]; and signed informed consent. Exclusion criteria were failure to complete the entire questionnaire and absence of signed informed consent. Withdrawal criteria were participant dropout and refusal to authorize dissemination of study results. Data collection began on 21 October 2024 and concluded on 20 September 2025 (Figure 1).

The original instrument was developed by Velázquez and Lovera in 2022 [24], and was created in Spanish, comprising 5 sections with 16 items. To address the objectives of this research, the instrument was adapted to integrate persistent clinical manifestations and post-infection illnesses. The adapted instrument currently contains 30 items organized into five sections: 1. sociodemographic data; 2. method of disease diagnosis; 3. time elapsed since symptom onset following disease; 4. pre-infection illnesses; and 5. clinical manifestations at the time of instrument administration and post-infection illnesses. Subsequently, a pilot test was conducted on 10% of the universe to measure internal consistency using Cronbach’s Alpha coefficient, obtaining a validity of 0.86, and reliability using the Kuder–Richardson coefficient (KR-20), with a value of KR-20 = 0.87. The questionnaire was administered during the 3rd and 4th weeks, three months after COVID-19 symptom onset.

The study procedure commenced with a detailed explanation of the research objectives to all participating nursing students, who were informed that the research was of minimal risk, and written informed consent was obtained, guaranteeing anonymous and confidential participation while protecting participant data in accordance with ethical research principles. Instructions regarding the questionnaire were provided. Data collection was conducted through a self-administered, in-person survey using a digital Google Forms format, sent to an institutional email address of the Autonomous University of the State of Mexico (UAEMéx), which provides data protection, with the researcher responsible for safeguarding the information [25].

Data analysis was performed using IBM SPSS version 27 software. Descriptive statistics were used, including measures of central tendency and dispersion to describe population characteristics. A bivariate analysis was conducted using RStudio version 4.4.1 (R Foundation for Statistical Computing, Vienna, Austria), using Fisher’s exact test (considering the assumptions of observed and expected frequencies) to identify significant associations between pain and both sociodemographic characteristics and clinical manifestations. A 95% confidence interval was considered, with statistical significance defined at *p* ≤ 0.05. A binary logistic regression model was performed to identify clinical manifestations associated with the presence of pain (1 = yes, 0 = no). The independent variables were sex, age, marital status, system-specific alterations, and clinical manifestations, as well as the interaction between them. Model fit was evaluated using the likelihood ratio (χ^2^ = 172.72, df = 14, *p* < 0.001), Nagelkerke R^2^ (0.581), and the Hosmer–Lemeshow test (χ^2^ = 16.13, df = 6, *p* = 0.013); AIC = 271.46. Statistical significance was set at *p* < 0.05.

## 3. Results

The clinical manifestations associated with pain in nursing students in emerging adulthood presenting with Long COVID are described below.

Table 1 presents the sociodemographic distribution of 307 (100.0%) nursing students in emerging adulthood with clinical manifestations of Long COVID. Age distribution showed that 154 (50.2%) were aged 18–20 years; 67 (21.8%) were 21–23 years; 76 (24.8%) were 24–26 years; and 10 (3.3%) were 27–29 years. The minimum age was 18 years, and the maximum was 29 years, with a range of 11 years. Mean age was 21 years (SD = 2.9), with a median of 20 and a mode of 18 years. Regarding sex, the majority were female, with 249 (81.1%), and 58 (18.9%) were male.

Regarding marital status, 286 (93.2%) were single; 6 (2.0%) were in common-law relationships; and 5 (1.6%) were married. Additionally, 9 (2.9%) were widowed, and 1 (0.3%) was divorced. By sex, among males, 55 (94.8%) were single, and 3 (5.2%) were in common-law relationships. Among females, 231 (75.2%) were single, 5 (1.6%) were married, 3 (1.0%) were in common-law relationships, 9 (2.9%) were widowed, and 1 (0.3%) was divorced (Table 1).

Table 2 describes the distribution of system-specific alterations reported by nursing students in emerging adulthood before and after COVID-19 infection. Following COVID-19 infection, system-specific alterations increased substantially, with 1747 (100.0%) clinical data points recorded by system, reflecting that nursing students experienced changes across multiple systems, including: neuropsychological 660 (37.8%), respiratory 466 (26.7%), sensory 331 (18.9%), urinary 144 (8.2%), gastrointestinal 94 (5.4%), musculoskeletal 48 (2.7%), cardiovascular 3 (0.2%), and immunological 1 (0.1%).

Table 3 describes the clinical manifestations that nursing students in emerging adulthood presented prior to COVID-19 infection. Among n = 307 (100.0%), 96 (31.2%) reported at least one clinical manifestation, including allergies 27 (28.1%); lupus 3 (3.1%); chronic gastritis 26 (27.1%); obesity 17 (17.7%); diabetes mellitus 4 (4.2%); chronic obstructive pulmonary disease (COPD) 7 (7.3%); asthma 6 (6.3%); systemic arterial hypertension 4 (4.2%); and heart failure 1 (1.0%). Among females, 76 (79.2%) reported illnesses and health alterations: chronic gastritis 25 (26.0%); allergies 18 (18.8%); obesity 12 (12.5%); COPD 7 (7.3%); asthma 4 (4.2%); systemic arterial hypertension 4 (4.2%); and lupus 3 (3.1%). Among males, 20 (6.5%); 9 (9.4%), reported allergies, and 5 (5.2%) obesity.

Following COVID-19 infection, nursing students reported 1747 clinical manifestations and persistence of pre-existing illnesses: dyspnea 307 (17.6%); dysgeusia 150 (8.6%); dysuria 140 (8.0%); anosmia 119 (6.8%); hypersomnia 96 (5.5%); headache 95 (5.4%); and fatigue 94 (5.3%). Among females, 1338 clinical data points were recorded (76.6% of total post-infection manifestations), indicating that participants reported multiple manifestations per individual like dyspnea 249 (14.3%); dysgeusia 112 (6.4%); dysuria 106 (6.1%); anosmia 87 (5.0%); headache 76 (4.4%); memory impairment 72 (4.1%); and fatigue 71 (4.1%). Males reported: dyspnea 58 (3.3%); dysgeusia 38 (2.2%); dysuria 34 (1.9%); and anosmia 32 (1.8%). Notably, 18 (1.0%) males reported decreased vision.

Table 4 describes the presence or absence of pain in relation to certain clinical manifestations in n = 307 nursing students in emerging adulthood with Long COVID. Overall, 124 (40.4%) reported the presence of pain, and 183 (59.6%) reported absence of pain. Among females, 97 (39.0%) reported pain and 152 (61.0%) did not. By age group, the 24–26-year group had the highest prevalence of pain with 49 (19.7%), followed by 21–23 years with 23 (9.2%), 18–20 years with 22 (8.8%), and 27–29 years with 3 (1.2%). Among males, 27 (46.6%) reported pain and 31 (53.4%) reported absence of pain. The age group with the highest pain prevalence was similar to females at 24–26 years with 11 (19.0%), followed by 21–23 years with 9 (15.5%), 18–20 years with 5 (8.6%), and 27–29 years with 2 (3.4%) cases. Chi-square testing showed a statistically significant association between age and pain (χ^2^ = 83.65; *p* < 0.001), which was significant only in the female group.

Table 5 describes pain-related clinical manifestations in nursing students in emerging adulthood with Long COVID clinical data; 198 (100.0%) pain manifestations were reported. Headache was the most frequent, with 95 (48.0%), followed by chest oppression 53 (26.8%), myalgias 46 (23.2%), and less frequently pulmonary pain and arthralgia, each with 2 (1.0%). By sex, 159 (80.3%) manifestations were reported by females, compared to 39 (19.7%) by males.

To identify the clinical manifestations associated with pain in nursing students in emerging adulthood with Long COVID, a Binary Logistic Regression model was estimated. The results summarize the goodness-of-fit statistics, coefficients, odds ratios, and the significance level of each variable. The dependent variable was the presence or absence of pain following infection (1 = yes, 0 = no). The model included as independent variables the sociodemographic characteristics (sex and age group) and persistent clinical manifestations present before and after COVID-19 infection.

Additionally, the interaction between sex and age group was incorporated, considering its epidemiological relevance for sex differences in a young population with Long COVID.

Clinically relevant variables (sex, age) were retained in the model regardless of their association with pain, while the remaining variables were selected based on their clinical plausibility and the results of the bivariate analysis.

The adjusted model was statistically significant. The model’s likelihood ratio showed that the included variables contributed to explaining the presence of pain compared to the null model (χ^2^ = 172.72, df = 14, *p* < 0.001); it explains a considerable proportion of the variation in the presence of pain (Nagelkerke R^2^ = 0.581). The Hosmer and Lemeshow test indicated a significant result, suggesting differences between the observed and expected values predicted by the model (χ^2^ = 16.13, df = 6, *p* = 0.013), which is attributable to the limitation of the sample size; and an Akaike Information Criterion of AIC = 271.46 (Table 6).

The model identified persistent clinical manifestations associated with the presence of pain, supporting the multisystemic nature of Long COVID: taste alteration (*p* < 0.001), fatigue (*p* = 0.048), decreased vision (*p* < 0.001), and gastrointestinal alteration (*p* = 0.025).

Regarding taste alteration (B = −2.353; OR = 0.10; 95% CI 0.03–0.32; *p* < 0.001), individuals presenting taste alteration were identified as 0.10 times less likely to report pain. Similarly, individuals reporting decreased vision showed a lower likelihood of presenting pain (B = −1.549; OR = 0.21; 95% CI 0.09–0.52; *p* < 0.001). These findings should be interpreted with caution, as they may reflect the existence of different clinical phenotypes within Long COVID, possible collinearity effects or symptom competition, as well as biases in self-report or symptom prioritization by participants.

In contrast, students with fatigue showed a higher likelihood of presenting pain (B = 0.946; OR = 2.58; 95% CI 1.01–6.58; *p* = 0.048), consistent with fatigue as a central manifestation of Long COVID, as did gastrointestinal alteration (B = 1.247; OR = 3.48; 95% CI 1.17–10.36; *p* = 0.025). These results demonstrate that the clinical manifestations with the greatest weight in explaining pain correspond primarily to gastrointestinal, neurological, and sensory manifestations.

No statistically significant differences in the presence of pain following infection were observed among participants according to sex, age, or their interaction (*p* > 0.05).

Collectively, these findings reinforce the complex and multisystemic nature of Long COVID and underscore the importance of considering both standardized measures and self-reports in its study, prioritizing those symptoms with greater clinical and statistical relevance in adjusted models.

## 4. Model Conclusions

The model confirms that the presence of pain in young adults with Long COVID is primarily associated with persistent manifestations of a multisystemic nature, particularly those of a neurological, gastrointestinal, and sensory type.

Taste alteration, fatigue, decreased vision, and gastrointestinal alteration stand out as significant symptoms, suggesting that certain subjective symptoms may provide relevant information, although their overall contribution to the model is limited compared to symptoms evaluated through structured instruments.

## 5. Discussion

This research identified the clinical manifestations associated with pain in a population of nursing students in emerging adulthood with Long COVID. Clinical manifestations were found both before and after COVID-19 infection: pre-infection manifestations were related to allergies, gastritis, and obesity, while post-infection manifestations included dyspnea, cough, dysgeusia, and dysuria. Notably, this study identified clinical manifestations in the urinary system, including recurrent urinary tract infections and kidney injury.

Age and sex were not found to be associated with pain; although age is not considered a determinant of the likelihood of pain, other studies, such as those by Shah DP and Oguz-Akarsu [26,27], have shown that persistent symptoms can occur significantly even at young ages. Furthermore, comparing these findings with the 2025 study by Shah DP, who investigated sex differences in prolonged COVID, greater risk of LC was reported in females aged 40–54 years, considering risk factors such as age, pregnancy, and menopause [26], in contrast to this research, which was conducted in a population with a younger age range, suggesting that the presence of pain could be lower in younger populations.

The results showed pain to be associated with several clinical manifestations, including gastrointestinal alterations, fatigue, decreased vision, and taste alteration, indicating that these manifestations play a role in the persistence of pain in this study population.

A study conducted by Selvakumar in 2023 found a limited difference between sexes in a cohort of adolescents and young adults, reporting the persistence of fatigue, headache, and cognitive alterations [28]. These results are comparable to the present study, where fatigue showed a significant association with pain.

Although females represented the largest proportion of the sample, this research found no statistically significant association with pain. These results differ from those described by D’Onofrio and Sékaly in their 2024 study, which indicated that sex differences in the response to SARS-CoV-2 are mediated by the interaction between the immune and endocrine systems, where estrogens enhance a more intense immune response, which could explain the greater predisposition of females to develop persistent symptoms, including pain-related manifestations associated with Long COVID [29]. This finding suggests that the effect of age on pain occurrence could differ by sex, particularly among older females within the group studied. Although this effect did not reach statistical significance, its magnitude suggests epidemiological relevance and warrants exploration in studies with a larger sample size, which could confirm or rule out this possibility.

In this regard, research conducted by Sudre C. et al. indicated that female sex, early headache, and fatigue are important characteristics of Long COVID [30], while Davis H. et al. reported a predominance of persistent symptoms in young females [31]. Al-Aly Z. et al. and Nalbandian et al. described that musculoskeletal pain and neurological and respiratory alterations are part of the persistent multisystemic manifestations following infection [31,32]. Similarly, Whitaker M. et al. identified that females and individuals with a greater number of initial symptoms presented a higher risk of developing Long COVID [33]. Based on the above, it can be stated that the presence of persistent clinical manifestations is associated with an increased probability of pain as a sequela of COVID-19, supporting the alternative hypothesis regarding the association of pain in nursing students in emerging adulthood with Long COVID.

Likewise, among the other manifestations associated with pain, gastrointestinal alteration—identified as a significant finding—is consistent with what is reported by Mohammed et al. (2024), who, through a systematic review, identified gastrointestinal complications following COVID-19 infection, highlighting inflammatory, vascular, and structural conditions, as well as nausea and abdominal pain [34]. These findings show that gastrointestinal involvement can persist, causing severe long-term complications.

Regarding taste alteration, this research identified a significant association with a lower likelihood of reporting pain. This result is consistent with what is reported by De Luca et al. (2022), who identified that taste alterations can persist as part of chemosensory dysfunction for a prolonged period after COVID-19 [35]; however, in this study, taste alteration was not associated with a higher likelihood of pain—rather, participants with this manifestation showed a lower likelihood of reporting pain.

Similarly, decreased vision was significantly associated with a lower likelihood of reporting pain, in contrast to what is described by Kaleem S. (2025), who reports ocular symptoms including blurred vision and vision loss, evidencing the persistence of visual alterations [36]; in this research, it was associated with a lower likelihood of reporting pain in this study population.

Finally, clinical manifestations associated with pain generate health alterations in a very young population, such as emerging adulthood; therefore, it is necessary to orient care toward early detection and management of manifestations, given their association with pain.

## 6. Conclusions

Clinical manifestations associated with Long COVID in nursing students following infection are related to persistent symptoms of a multisystemic nature, particularly those of a neurological, gastrointestinal, and sensory type; fatigue and gastrointestinal alterations were associated with a higher likelihood of pain among nursing students in emerging adulthood following infection. Overall, these findings demonstrate that Long COVID can affect nursing students in emerging adulthood at a multisystemic level.

## 7. Study Limitations

This study is relevant, particularly for including a sample of nursing students in emerging adulthood; however, there are limitations related to the following: the male population is small, which limits the ability to affirm a significant association based on the results; this could be considered for future studies using sex-matched samples. The study was conducted at a single institution in the State of Mexico, and although it includes the largest number of students enrolling in nursing studies, it was not possible to include additional institutions. This study did not measure pain to identify symptom severity, as the objective was to identify clinical manifestations in relation to the presence or absence of pain in nursing students; this therefore remains as a proposal for future research to continue strengthening the epistemic aspects related to pain and Long COVID. Future research is suggested that considers variables such as high-sensitivity C-reactive protein, vaccination status, and possible reinfection, given that pain in LC presents a multifactorial and multisystemic pattern.

Finally, as this is a disease that emerged in 2020, previous studies exist in specific communities, with limitations identified for the 18–29 year age group; this highlights the importance of studying emerging adulthood, as it is the age at which diseases that may lead to chronicity are triggered.

## Figures and Tables

**Table 1:** Nursing students in emerging adulthood with Long COVID by age and marital status according to sex.

Variable	Sex				Total	
	Female		Male			
	*n* = 249 (100.0%)		*n* = 58 (100.0%)		307 (100.0%)	
	n	%	n	%	n	%
Age						
18–20	135	54.2	19	32.8	154	50.2
21–23	52	20.9	15	25.9	67	21.8
24–26	57	22.9	19	32.8	76	24.8
27–29	5	2	5	8.6	10	3.3
Total	249	100	58	100	307	100
Marital Status						
Single	231	92.8	55	94.8	286	93.2
Common-law relationship	3	1.2	3	5.2	6	2
Married	5	2	---	---	5	1.6
Widowed	9	3.6	---	---	9	2.9
Divorced	1	0.4	---	---	1	0.3
Total	249	100	58	100	307	100

Source: Data obtained from the modified questionnaire on post-SARS-CoV-2 health conditions, 2025.

**Table 2:** System-specific alterations in nursing students in emerging adulthood before and after COVID-19 infection according to sex.

System-Specific Alterations	Pre-Infection						Post-Infection					
	Sex											
	Female		Male		Total		Female		Male		Total	
	f	%	f	%	f	%	f	%	f	%	f	%
Neuropsychological	-	-	-	-	-	-	512	29.3	148	8.5	660	37.8
Cardiovascular	4	4.2	1	1	5	5.2	-	-	3	0.2	3	0.2
Respiratory	11	11.5	2	2.1	13	13.5	372	21.3	94	5.4	466	26.7
Endocrine-metabolic	15	15.6	7	7.3	22	22.9	-	-	-	-	-	-
Gastrointestinal	25	26	1	1	26	27.1	71	4.1	23	1.3	94	5.4
Urinary	-	-	-	-	-	-	110	6.3	34	1.9	144	8.2
Immunological	21	21.9	9	9.4	30	31.3	1	0.1	-	-	1	0.1
Sensory	-	-	-	-	-	-	232	13.3	99	5.7	331	18.9
Musculoskeletal	-	-	-	-	-	-	40	2.3	8	0.5	48	2.7
Total	76	79.2	20	20.8	96	100	1338	76.6	409	23.4	1747	100

Source: Data obtained from the modified questionnaire on post-SARS-CoV-2 health conditions, 2025. Note: Frequencies and percentages do not sum to the total sample as participants could present more than one clinical manifestation following infection.

**Table 3:** Clinical manifestations and diseases in nursing students in emerging adulthood before and after COVID-19 infection.

Clinical Manifestations and Diseases	Pre-Infection						Post-Infection					
	Sex						Sex					
	Female		Male		Total		Female		Male		Total	
	n = 76		n = 20		n = 96		n = 1338		n = 409		n = 1747	
	f	%	f	%	f	%	f	%	f	%	f	%
Depression	-	-	-	-	-	-	24	1.4	9	0.5	33	1.9
Fatigue	-	-	-	-	-	-	71	4.1	23	1.3	94	5.3
Dizziness	-	-	-	-	-	-	49	2.8	7	0.4	56	3.2
Memory impairment	-	-	-	-	-	-	72	4.1	20	1.1	92	5.3
Impaired attention	-	-	-	-	-	-	64	3.7	14	0.8	78	4.5
Migraine	-	-	-	-	-	-	3	0.2	1	0.1	4	0.2
Insomnia	-	-	-	-	-	-	31	1.8	11	0.6	42	2.4
Hypersomnia	-	-	-	-	-	-	70	4.0	26	1.5	96	5.5
Behavioral alterations	-	-	-	-	-	-	49	2.8	16	0.9	65	3.7
Tinnitus	-	-	-	-	-	-	1	0.1	-	-	1	0.1
Anxiety	-	-	-	-	-	-	2	0.1	2	0.1	4	0.2
Systemic arterial hypertension	4	4.2	-	-	4	4.2	-	-	3	0.2	3	0.2
Heart failure	-	-	1	1	1	1	-	-	-	-	-	-
Asthma	4	4.2	2	2.1	6	6.3	-	-	-	-	-	-
Chronic obstructive pulmonary disease (COPD)	7	7.3	-	-	7	7.3	-	-	-	-	-	-
Dyspnea	-	-	-	-	-	-	249	14.3	58	3.3	307	17.6
Cough	-	-	-	-	-	-	55	3.1	17	1.0	72	4.1
Sputum	-	-	-	-	-	-	25	1.4	4	0.2	29	1.7
Diabetes mellitus	3	3.1	1	1	4	4.2	-	-	-	-	-	-
Dyslipidemia	-	-	1	1	1	1	-	-	-	-	-	-
Obesity	12	12.5	5	5.2	17	17.7	-	-	-	-	-	-
Gastritis	25	26	1	1	26	27.1	41	2.3	14	0.8	55	3.1
Irritable bowel	-	-	-	-	-	-	5	0.3	-	-	5	0.3
Diarrhea	-	-	-	-	-	-	3	0.2	-	-	3	0.2
Constipation	-	-	-	-	-	-	1	0.1	-	-	1	0.1
Gastric reflux	-	-	-	-	-	-	0	0.0	1	0.1	1	0.1
Colitis	-	-	-	-	-	-	21	1.2	8	0.5	29	1.7
Dysuria	-	-	-	-	-	-	106	6.1	34	1.9	140	8.0
UTI	-	-	-	-	-	-	2	0.1	-	-	2	0.1
Polyuria	-	-	-	-	-	-	1	0.1	-	-	1	0.1
Acute kidney injury	-	-	-	-	-	-	1	0.1	-	-	1	0.1
Allergies	18	18.8	9	9.4	27	28.1	1	0.1	-	-	1	0.1
Lupus	3	3.1	-	-	3	3.1	-	-	-	-	-	-
Headache	-	-	-	-	-	-	76	4.4	19	1.1	95	5.4
Hearing loss	-	-	-	-	-	-	15	0.9	11	0.6	26	1.5
Decreased vision	-	-	-	-	-	-	18	1.0	18	1.0	36	2.1
Anosmia	-	-	-	-	-	-	87	5.0	32	1.8	119	6.8
Dysgeusia	-	-	-	-	-	-	112	6.4	38	2.2	150	8.6
Throat irritation	-	-	-	-	-	-	-	-	3	0.2	3	0.2
Chest oppression	-	-	-	-	-	-	41	2.3	12	0.7	53	3.0
Myalgia	-	-	-	-	-	-	38	2.2	8	0.5	46	2.6
Pulmonary pain	-	-	-	-	-	-	2	0.1	-	-	2	0.1
Arthralgia	-	-	-	-	-	-	2	0.1	-	-	2	0.1
Total	76	79.2	20	20.8	96	100	1338	76.6	409	23.4	1747	100.0

Source: Data obtained from the modified questionnaire on post-SARS-CoV-2 health conditions, 2025. Note: Frequencies and percentages do not sum to the total sample as participants could present more than one clinical manifestation following infection.

**Table 4:** Presence and absence of pain in nursing students in emerging adulthood with Long COVID according to sex and age groups.

Sex/Age										
	18–20		21–23		24–26		27–29		Total	
	f	%	f	%	f	%	f	%	f	%
**Female (n = 249)**										
Presence of pain	22	8.8	23	9.2	49	19.7	3	1.2	97	39.0
Absence of pain	113	45.4	29	11.6	8	3.2	2	0.8	152	61.0
Total	135	54.2	52	20.9	57	22.9	5	2.0	249	100.0
**Male (n = 58)**										
Presence of pain	5	8.6	9	15.5	11	19.0	2	3.4	27	46.6
Absence of pain	14	24.1	6	10.3	8	13.8	3	5.2	31	53.4
Total	19	32.8	15	25.9	19	32.8	5	8.6	58	100.0
**Total (n = 307)**										
Presence of pain	27	8.8	32	10.4	60	19.5	5	1.63	124	40.4
Absence of pain	127	41.4	35	11.4	16	5.2	5	1.63	183	59.6
Total	154	50.2	67	21.8	76	24.8	10	3.26	307	100.0

Source: Data obtained from the modified questionnaire on post-SARS-CoV-2 health conditions, 2025. Note: Participants presented more than one clinical manifestation following COVID-19 infection; of these, 124 (40.4%) nursing students reported pain. Participants were calculated within the combination of sex and age (x^2^ = 83.65; *p*< 0.001).

**Table 5:** Pain-related clinical manifestations reported by nursing students in emerging adulthood with Long COVID according to sex.

Clinical Manifestation	Sex				Total	
	Female		Male			
	f	%	f	%	f	%
Headache	76	38.4	19	9.6	95	48.0
Chest oppression	41	20.7	12	6.1	53	26.8
Myalgia	38	19.2	8	4.0	46	23.2
Pulmonary pain	2	1.0	-	-	2	1.0
Arthralgia	2	1.0	-	-	2	1.0
Total	159	80.3	39	19.7	198	100.0

Source: Data obtained from the modified questionnaire on post-SARS-CoV-2 health conditions, 2025. Note: The 124 participants reported more than one pain-related manifestation; therefore, 198 manifestations were recorded, and the sum of percentages equals 100%.

**Table 6:** Clinical manifestations associated with pain in nursing students in emerging adulthood with Long COVID.

Variable	B (log-OR)	Standard Error	95% IC	*p*-Value
Sex				
Male *				
Female vs. Male	−0.659	0.633	0.52 (0.15–1.79)	0.298
Age Group				
18–20 *				
21–23 vs. 18–20	−0.742	0.965	0.48 (0.07–3.16)	0.442
24–26 vs. 18–20	−1.129	0.913	0.32 (0.05–1.94)	0.216
27–29 vs. 18–20	−2.035	1.609	0.13 (0.01–3.06)	0.206
Clinical Manifestations (reported by students)				
Taste alteration	−2.353	0.623	0.10 (0.03–0.32)	<0.001
Persistent clinical manifestations				
Sputum	−0.530	0.579	0.59 (0.19–1.83)	0.360
Sleep alteration due to breathing	−0.060	0.547	0.94 (0.32–2.75)	0.913
Fatigue	0.946	0.479	2.58 (1.01–6.58)	0.048
Decreased vision	−1.549	0.455	0.21 (0.09–0.52)	<0.001
Memory impairment	0.838	0.463	2.31 (0.93–5.74)	0.070
Gastrointestinal alteration	1.247	0.557	3.48 (1.17–10.36)	0.025
Sex × Age Interaction				
Female × 21–23 years	−0.108	1.008	0.90 (0.12–6.48)	0.915
Female × 24–26 years	1.397	0.968	4.04 (0.61–26.95)	0.149
Female × 27–29 years	2.735	2.024	15.41 (0.29–813.70)	0.176

Source: Data obtained from the modified questionnaire on post-SARS-CoV-2 health conditions, 2025 Note: The dependent variable was the presence or absence of pain following infection (1 = yes, 0 = no). Categorical variables are interpreted relative to their reference category; age was included as an ordinal categorical variable. B (log-OR); SE = Standard Error; OR = odds ratio; 95% CI = 95% confidence interval; *p* = *p*-value. The model included n = 307 participants. Model fit: χ^2^ = 172.72, df = 14, *p* < 0.001; Nagelkerke R^2^ = 0.581; Hosmer–Lemeshow test: χ^2^ = 16.13, df = 6, *p* = 0.013. Reference categories correspond to: sex = male *, age group = 18–20 years *, absence of taste alteration *, absence of sputum *, absence of sleep alteration due to breathing *, absence of fatigue*, absence of decreased visual acuity*, absence of memory impairment*, and absence of gastrointestinal alterations *.

## Data Availability

Data availability is contained in an Excel database and is not yet in the public domain due to privacy concerns of the participants. Data are under the safeguard of the researchers in the UAEMéx cloud storage.

## Public Involvement Statement

Public involvement was limited to nursing students who participated by signing informed consent and attending nursing consultation to complete the questionnaire.

## Guidelines and Standards Statement

This study was written following the STROBE guidelines for cross-sectional studies. Additionally, the manuscript was structured according to the IMRD format (Introduction, Methods, Results, and Discussion) [37].

## References

[1] World Health Organization COVID-19 Epidemiological Update—15 July 2024. https://www.who.int/publications/m/item/covid-19-epidemiological-update-edition-169.

[2] Pizzato M., Gerli A.G., Vecchia C.L., Alicandro G. (2024). Impact of COVID-19 on total excess mortality and geographic disparities in Europe, 2020–2023: A spatio-temporal analysis. Lancet Reg. Health–Eur..

[3] Dirección de Vigilancia Epidemiológica de Enfermedades Transmisibles. Informe Semanal. https://www.gob.mx/cms/uploads/attachment/file/1088895/Informesemanal_ERV_SE26_2026_06.07.2026_.pdf.

[4] Siddiqi H.K., Mehra M.R. (2020). COVID-19 illness in native and immunosuppressed states: A clinical–therapeutic staging proposal. J. Heart Lung Transpl..

[5] Staffolani S., Iencinella I., Cimatti M., Tavio M. (2022). Long COVID-19 syndrome as a fourth phase of SARS-CoV-2 infection. Infez. Med..

[6] Iqbal N.T., Khan H., Khalid A., Mahmood S.F., Nasir N., Khanum I., de Siqueira I., Van Voorhis W. (2025). Chronic inflammation in post-acute sequelae of COVID-19 modulates gut microbiome: A review of literature on COVID-19 sequelae and gut dysbiosis. Mol. Med..

[7] Nalbandian A., Sehgal K., Gupta A., Madhavan M.V., McGroder C., Stevens J.S., Cook J.R., Nordvig A.S., Shalev D., Sehrawat T.S. (2021). Post-acute COVID-19 syndrome. Nat. Med..

[8] Fernández-de-las-Peñas C., Nijs J., Neblett R., Polli A., Moens M., Goudman L., Shekhar Patil M., Knaggs R.D., Pickering G., Arendt-Nielsen L. (2022). Phenotyping Post-COVID Pain as a Nociceptive, Neuropathic, or Nociplastic Pain Condition. Biomedicines.

[9] National Academies of Sciences, Engineering, and Medicine (2024). Defining Long COVID: A State of Chronic and Systemic Disease with Profound Consequences.

[10] Raja S.N., Carr D.B., Cohen M., Finnerup N.B., Flor H., Gibson S., Keefe F.J., Mogil J.S., Ringkamp M., Sluka K.A. (2020). The revised International Association for the Study of Pain definition of pain: Concepts, challenges, and compromises. Pain.

[11] Cohen S.P., Vase L., Hooten W.M. (2021). Chronic pain: An update on burden, best practices, and new advances. Lancet.

[12] Caronna E., Ballvé A., Llauradó A., Gallardo V.J., Ariton A.M., Lallana S., Lopez Maza S., Olive Gadea M., Quibus L., Restrepo J.L. (2020). Headache: A striking prodromal and persistent symptom, predictive of COVID-19 clinical evolution. Cephalalgia.

[13] Membrilla J.A., Caronna E., Trigo-López J., González-Martínez A., Layos-Romero A., Pozo-Rosich P., Guerrero-Peral Á., Gago-Veiga A.B., Andrés-López A., de Terán J.D. (2021). Persistent headache after COVID-19: Pathophysiology, clinic and treatment. Neurol. Perspect..

[14] García-Azorín D., Layos-Romero A., Porta-Etessam J., Membrilla J.A., Caronna E., Gonzalez-Martinez A., Mencia Á.S., Segura T., Gonzalez-García N., Díaz-de-Terán J. (2022). Post-COVID-19 persistent headache: A multicentric 9-months follow-up study of 905 patients. Cephalalgia.

[15] Sunkersing D., Goodfellow H., Mu Y., Ramasawmy M., Murali M., Adams L., FitzGerald T.J., Blandford A., Stevenson F., Bindman J. (2024). Long COVID symptoms and demographic associations: A retrospective case series study using healthcare application data. JRSM Open.

[16] González González F., Cortés Correa C., Peñaranda Contreras E. (2021). Cutaneous manifestations in patients with COVID-19: Clinical characteristics and postulated pathophysiological mechanisms. Actas Dermo-Sifiliogr..

[17] Becerra-Canale B., Campos-Martínez H.M.M., Campos-Sobrino M., Aquije-Cárdenas G.A. (2022). Post-traumatic stress and quality of life of post-COVID-19 patients in primary care. Aten. Primaria.

[18] Faro M., Sàez-Francàs N., Castro-Marrero J., Aliste L., Fernández de Sevilla T., Tomás J. (2016). Gender differences in chronic fatigue syndrome. Reumatol. Clin..

[19] Bai F., Tomasoni D., Falcinella C., Barbanotti D., Castoldi R., Mulè G., Augello M., Mondatore D., Allegrini M., Cona A. (2022). Female gender is associated with long COVID syndrome: A prospective cohort study. Clin. Microbiol. Infect..

[20] Kompaniyets L., Bull-Otterson L., Boehmer T.K., Baca S., Alvarez P., Hong K., Hsu J., Harris A.M., Gundlapalli A.V., Saydah S. (2022). Post-COVID-19 Symptoms and Conditions Among Children and Adolescents—United States, March 1, 2020–January 31, 2022. MMWR Morb. Mortal. Wkly. Rep..

[21] Arnett J.J., Žukauskiene R., Sugimura K. (2014). The new life stage of emerging adulthood at ages 18–29 years: Implications for mental health. Lancet Psychiatry.

[22] Winpenny E.M., Smith M., Penney T., Foubister C., Guagliano J.M., Love R., Clifford Astbury C., van Sluijs E.M., Corder K. (2020). Changes in physical activity, diet, and body weight across the education and employment transitions of early adulthood: A systematic review and meta-analysis. Obes. Rev..

[23] Li D., Zou L., Zhang Z., Zhang P., Zhang J., Fu W., Mao J., Cao S. (2021). The Psychological Effect of COVID-19 on Home-Quarantined Nursing Students in China. Front. Psychiatry.

[24] Velázquez G., Lovera G. (2022). Evaluation of clinical condition and quality of life following coronavirus infection. Rev. Argent. Med..

[25] Cisneros Caicedo A.J., Guevara García A.F., Urdánigo Cedeño J.J., Garcés Bravo J.E. (2022). Techniques and instruments for data collection that support scientific research in times of pandemic. Dominio Cienc..

[26] Shah D.P., Thaweethai T., Karlson E.W., Bonilla H., Horne B.D., Mullington J.M., Wisnivesky J.P., Hornig M., Shinnick D.J., Klein J.D. (2025). Sex Differences in Long COVID. JAMA Netw. Open.

[27] Oguz-Akarsu E., Gullu G., Kilic E., Dinç Y., Akdag G., Rehber C., Karli N. (2024). Beyond the acute: Pain in long COVID survivors at 1.5 years. Neurol. Sci..

[28] Selvakumar J., Havdal L.B., Drevvatne M., Brodwall E.M., Lund Berven L., Stiansen-Sonerud T., Einvik G., Leegaard T.M., Tjade T., Michelsen A.E. (2023). Prevalence and Characteristics Associated with Post–COVID-19 Condition Among Nonhospitalized Adolescents and Young Adults. JAMA Netw. Open.

[29] D’Onofrio V., Sékaly R.P. (2024). The immune–endocrine interplay in sex differential responses to viral infection and COVID-19. Trends Immunol..

[30] Sudre C.H., Murray B., Varsavsky T., Graham M.S., Penfold R.S., Bowyer R.C., Pujol J.C., Klaser K., Antonelli M., Canas L.S. (2021). Attributes and predictors of long COVID. Nat. Med..

[31] Davis H.E., Assaf G.S., McCorkell L., Wei H., Low R.J., Re’em Y., Redfield S., Austin J.P., Akrami A. (2021). Characterizing long COVID in an international cohort: 7 months of symptoms and their impact. EClinicalMedicine.

[32] Al-Aly Z., Xie Y., Bowe B. (2021). High-dimensional characterization of post-acute sequelae of COVID-19. Nature.

[33] Whitaker M., Elliott J., Chadeau-Hyam M., Riley S., Darzi A., Cooke G., Ward H., Elliott P. (2022). Persistent COVID-19 symptoms in a community study of 606,434 people in England. Nat. Commun..

[34] Mohammed I., Podhala S., Zamir F., Shiyam S., Salameh A.R., Salahuddin Z., Salameh H., Kim C., Sinan Z., Kim J. (2024). Gastrointestinal Sequelae of COVID-19: Investigating Post-Infection Complications—A Systematic Review. Viruses.

[35] De Luca P., Di Stadio A., Colacurcio V., Marra P., Scarpa A., Ricciardiello F., Cassandro C., Camaioni A., Cassandro E. (2022). Long COVID, audiovestibular symptoms and persistent chemosensory dysfunction: A systematic review of the current evidence. Acta Otorhinolaryngol. Ital..

[36] Kaleem S., Sawano M., Arun A.S., Warner F., Zhou T., Huang C., Bhattacharjee B., Lu Y., Iwasaki A., Nwanyanwu K. (2026). Ocular symptoms in long COVID: A cross-sectional study. Clin. Ophthalmol..

[37] von Elm E., Altman D.G., Egger M., Pocock S.J., Gøtzsche P.C., Vandenbroucke J.P. (2007). The Strengthening the Reporting of Observational Studies in Epidemiology (STROBE) statement: Guidelines for reporting observational studies. Lancet.

